# Insulin-Like-Growth-Factor-Binding-Protein-3 (IGFBP-3) Contrasts Melanoma Progression *In Vitro* and *In Vivo*

**DOI:** 10.1371/journal.pone.0098641

**Published:** 2014-06-06

**Authors:** Antimo Naspi, Vincenzo Panasiti, Franco Abbate, Vincenzo Roberti, Valeria Devirgiliis, Michela Curzio, Martina Borghi, Francesco Lozupone, Simone Carotti, Sergio Morini, Eugenio Gaudio, Stefano Calvieri, Paola Londei

**Affiliations:** 1 Istituto Pasteur-Fondazione Cenci-Bolognetti, Dpt. Biotecnologie Cellulari ed Ematologia, University of Rome Sapienza, Rome, Italy; 2 Plastic Surgery Unit, Campus Bio-Medico University of Rome, Rome, Italy; 3 Department of Internal Medicine and Medical Specialties, University of Rome Sapienza, Rome, Italy; 4 Department of Therapeutic Research and Medicine Evaluation, Unit of Antitumor Drugs, Istituto Superiore di Sanita', Rome, Italy; 5 Department of Human Anatomy (CIR), University Campus Bio-Medico of Rome, Rome, Italy; 6 Department of Human Anatomy, University of Rome Sapienza, Rome, Italy; IDI, Istituto Dermopatico dell'Immacolata, Italy

## Abstract

Insulin-like-factor-binding-protein 3 (IGFBP-3) is known to modulate the activity of insulin-like growth factors (IGFs) besides having a number of IGF-independent effects on cell growth and survival. IGFBP-3 has been reported to decrease significantly in the blood serum of patients affected by certain cancers. In the present work, we have evaluated the levels of IGFBP-3 in the blood serum and tissues of patients affected by cutaneous melanoma, showing that loss of IGFBP-3 from both is strongly correlated with disease progression and reduced survival. *In vitro* treatment with IGFBP-3 of human and murine metastatic melanoma cell lines specifically inhibited the cells' migratory and invasive behaviour, inducing up-regulation of melanocytic differentiation markers such as tyrosinase activity and melanin content. A molecular analysis of the cellular pathways transducing the effect of IGFBP-3 implicated the Akt-GSK3β axis. Moreover, administration of IGFBP-3 *in vivo* to SCID mice inoculated with human metastatic melanoma cells strongly reduced or completely inhibited tumor growth. In summary, IGFBP-3 appears to exert a specific inhibitory effect on melanoma growth and dissemination, suggesting that it may qualify as a useful therapeutic agent in melanomas and perhaps other cancers, at the least as a valid adjuvant therapy during treatment with conventional anti-tumoral drugs.

## Introduction

Melanoma is an aggressive malignancy whose incidence is increasing worldwide. Actually, much of this increase could depend on the higher frequency of early diagnosis; however, from 1990 to 2002 the mortality rate has decreased by only 0.3% per year, mainly because there are no standard systemic therapies to improve the survival of stage-IV melanoma patients [1]–[2].

Rather than as a single disease, melanoma should be viewed as a heterogeneous cluster of disorders with defects influencing important cellular processes such as cell cycle regulation, cell signalling pathways, cell adhesion, cell differentiation and cell death [3]. This heterogeneity in molecular faults emphasizes the need for individualisation of melanoma diagnosis, prognosis and treatment.

On the basis of the American Joint Committee on Cancer (AJCC) staging system (TNM), current prognostic biomarkers in melanoma are represented by Breslow tumour thickness, presence of ulceration, mitotic rate, and extent of nodal involvement for primary cutaneous melanoma, serum lactate dehydrogenase (LDH) and site of metastases [4]. More research is needed to identify other diagnostic and prognostic molecular markers that could open possibilities for achieving better and more personalised treatments.

The Insulin-like Growth Factors (IGFs) system comprises IGF1, IGF2, the IGF receptors, and the IGF binding proteins (IGFBPs), which regulate the bioavailability of insulin and IGFs [5]. IGF family proteins are involved in proliferation and apoptosis, and thus play a significant role on growth of both normal and malignant cells [3]. In the circulation, about 90% of IGF1 is bound to IGFBP-3, [4]. In addition, IGFBP-3 exerts anti-proliferative and apoptotic effects that are mediated through a specific cell surface receptor [5]. Epidemiological studies show that high levels of IGF1 and low levels of IGFBP-3 are associated with an increased risk for several common cancers, including prostate, breast, lung, and colorectal cancer [6]–[8].

Deregulation of the IGF system is a common pattern in malignancy [6]–[9]; therefore IGFs/IGFBPs might represent tumour markers useful both for diagnosis and follow up [10]–[11].

IGF-binding-protein 3 (IGFBP-3) is the best-known member of the IGFBP family. Many studies have shown its capacity to inhibit proliferation of breast, lung and prostate cancer cells [12]–[14]. In a previous report, we have shown that a strong correlation exists between the serum concentration of full-size, glycosylated IGFBP-3 and disease progression in melanoma patients [15]. In this study, we have investigated the effect of administering recombinant IGFBP-3 to cell cultures from primary and metastatic melanoma, from both human and murine sources. We found that IGFBP-3 strongly inhibited the migratory and invasive behaviour of malignant cells, moreover inducing up-regulation of certain melanocytic differentiation markers. These effects of IGFBP-3 are independent of IGF-1 and are transduced at the molecular level through the Akt-GSK3β pathway. Finally, we show that recombinant human IGFBP-3 is also able to strongly reduce melanoma growth in mouse models *in vivo*.

## Methods

From August 2007 to November 2010, 48 patients affected by cutaneous melanoma, followed as in-patients and/or out-patients in the Department of Dermatology of the University “Sapienza” of Rome, were enrolled. Recorded variables were: age, sex, date of diagnosis, melanoma histotype, Breslow thickness, Clark level, ulceration, and stage disease in agreement with the AJCC 2009 staging system [4].

For patients with measurable metastatic lesions, metastatic volume was calculated from radiologic imaging obtained by helicoidally computed tomography scan (CT) according to the RECIST guideline [16]. Metastatic lesions were accurately measured in at least one dimension (longest diameter in the plane of measurement was considered) with a minimum size of 10 mm by CT scan, to categorize the tumour lesions and lymph nodes as either measurable or non-measurable. Each measurable metastasis was considered as a spherical formation whose diameter was the average of the minimum diameter and maximum diameter. The volumes calculated using the following formula: volume  =  4/3 π r^3^.

Blood samples were taken from each patient when first seen. For 28 of them a second blood sample was taken during the follow up. The samples were collected, de-identified, random numbered and stored at −80°C until analyzed.

For each subject, a written consent had been obtained to perform analyses either on blood or melanoma tissue. In compliance with the Helsinki Declaration, the research programs had been approved by the ethics committee of Policlinico Umberto I of Rome.

IGF-1 and IGFBP-3 were measured in the serum samples of all the enrolled patients.

### ELISA/RIA

IGF-1 was measured by radioimmunoassay after acid-ethanol extraction using kits from DIAsource ImmunoAssays S.A, (Belgium). All measurements were performed in duplicate.

IGFBP-3 was measured by enzyme-linked immunosorbent assays (ELISA) from Diagnostic Systems Laboratories (DSL, Webster, TX, USA). All measurements were performed in duplicate.

Furthermore, IGF-1 and IGFBP-3 values were age and sex adjusted [17].

The IGF-1/IGFBP-3 molar ratio was calculated based on 1 ng/mL IGF-1  =  0.130 nmol IGF-1 and 1 ng/mL IGFBP-3  =  0.036 nmol IGFBP-3.

### Immuno-Histochemistry and Immuno-Fluorescence

Samples of primary melanoma (20) and dermal metastases (20) were analyzed.

Immunohistochemistry was performed on 3–5 µm thick sections obtained from formalin-fixed tissue embedded in paraffin. Antigen retrieval was performed using a PT module (pH 6; Thermo Fisher Scientific, Fremont, CA, USA). The immuno-histochemical procedure was performed according to the alkaline phosphatase method. Briefly, rabbit polyclonal anti-MMP-9 antibody (Thermo Scientific, RB-9234-P) diluted 1∶200 and rabbit polyclonal anti-IGFBP-3 antibody (AbD, 5345-5109) diluted 1∶100 were used as the primary antibody. After Tris-phosphate buffered saline (TBS) rinse, sections were further incubated with labelled polymer in accordance with the standard UltraVision AP detection system (Thermo Fisher Scientific). After a final TBS rinse, all immuno-histochemical reactions were visualized by using Liquid Fast-Red Substrate System (Thermo Scientific, Runcorn, UK) as the chromogen and hematoxylin as the counterstain.

The immuno-histochemical evaluation was performed independently by 2 researchers without knowledge of the patient's data, using a double-headed microscope. Inter-observer agreement was higher than 90%. The number of positive cells was separately counted for IGFBP-3 and MMP-9 under a light microscope at 200X magnification. For each slide, at least 7–10 microscopic fields were randomly chosen.

A five grade-semi-quantitative scoring system (score 0–4) was adopted for the evaluation of IGFBP-3 and MMP-9 immuno-histochemical expression. The score was graded according to the percentage of stained cells: score 0 was defined as the presence of stained positive cells ≤ 5%, score 1, 2 and 3 were defined as the presence respectively of 6% to 25%, 26% to 50% and 51%- to 75% positive cells. Score 4 was defined as the presence of > 75% positive cells.

The immuno-histochemical score was evaluated separately for melanocytic and stromal cells and expressed as tumour and stromal score respectively. A total score was derived for each sample as the sum of tumour and stromal score.

For the identification of proliferating cells in tumours of xenografted SCID mice, the primary antibody used was anti- Ki-67 (dilution 1/100; mouse monoclonal, clone MIB-1, Dako, Glostrup, Denmark) with 1 h of incubation. The immuno-histochemical procedure was performed using the MACH 1 Universal HRP-Polymer Detection kit (Biocare LLC, Concord, CA, USA) and Betazoid DAB (Biocare LLC, Concord, CA, USA) as chromogen. The proliferation index was determined as a percentage of proliferating cells (Ki-67 positive cells) in a population of about 800 cells. For this purpose two representative fields were randomly selected in each section and blind-counted by two independent observers.

### Cell Lines and Culture Conditions

The Wistar primary melanoma (WM) cell lines (BRAF V600E; NRAS wt) were kindly provided by Dr. Meenhard Herlyn, Wistar Institute, Philadelphia, PA. [18]. The cell lines Me501 (BRAF wt; NRAS G10D) were established from metastases surgically excised from melanoma patients at the Istituto Nazionale dei Tumori, Milan, Italy [19]–[20].

The murine melanoma B16F10 cell line (ATCC CRL6475™; BRAF wt; NRAS wt) was purchased from ATCC, Manassas, VA, USA [21].

All melanoma cell lines were seeded in 3-cm Petri dishes (2×10^5^ or 4×10^5^ per dish) in RPMI 1640 supplemented with 100 IU/mL penicillin, 100 µg/mL streptomycin (Life Technologies, Gaithersburg, MD), and 2 mmol/L glutamine (Life Technologies) with 10% FCS in a 5% CO_2_ environment at 37°C.

### Phospor-Proteome Determinations and Western Blot Analysis

For phosphor-proteome determinations, Proteome Profiler™ kits (Cat. N. ARY003, R&D Systems Europe Ltd.) were employed, following the firm's protocol. The intensity of the spots was measured densitometrically and quantified using the internal standards in the kits. The assays were perfomed in quadruplicate.

For western blot analyses of IGF-1 presence, IGF1-receptor phoshorylation state, Akt phosphorylation state and tyrosinase amounts, the cells were grown to 80% confluence and then harvested on ice using cell lysis buffer (20 mM Tris, pH 7.5, 150 mM NaCl, 1% Triton X, 1X protease and phosphatase inhibitor mixture (Roche Applied Science). Cells were dounce-homogenized and centrifuged at 10,000/*g* for 10 min. The supernatant was quantified using the Bradford assay; 30 µg of each lysate were run on 12.5% SDS-polyacrylamide gels under reducing conditions and transferred onto 0.2-μm nitrocellulose. Depending on the experiment, the membranes were probed with the following antibodies: rabbit polyclonal anti-phospho-Akt (ser 473); anti-total Akt antibodies; anti-IGF-1 receptor β, total; anti-IGF-1 receptor β (pTyr1135) (Cell Signaling); β-Tubulin (Sigma-Aldrich); rabbit monoclonal anti-tyrosinase antibodies (Epitomics); anti-IGF-1 rabbit monoclonal antibodies (Epitomics); anti-IGFBP-3 rabbit polyclonal antibodies (Acris). The reactions were visualized using the ECL system (Pierce) and quantified densitometrically when specified. All experiments were performed at least in triplicate.

### Gelatin Substrate Zymography

For gelatin zymography, 20 µL of serum-free medium were separated on 10% SDS-polyacrylamide gels containing 1 mg/mL bovine gelatin (Sigma, Deisenhofen, Germany) under non-reducing condition. Following electrophoresis, the gels were washed twice for 30 min in 2.5% Triton X-100 to remove SDS. After equilibration in enzyme substrate buffer (50 mM Tris-HCl, pH 7.5; 150 mM NaCl; 5 mM CaCl_2_, the gels were incubated in the same buffer overnight at 37°C. They were then stained with Coomassie Blue R 250 and destained in water. The experiments were performed in triplicate.

### IGFBP-3 Protease Assay

To assay for protease activity in the culture media of melanoma cells, 20 pmol of full-length, glycosylated IGFBP-3 was mixed with 20 µL of serum-free medium from Me501 cells and the samples were incubated at 37°C for 96 h either in the absence or in the presence of protease inhibitors (Protease-Inhibitor-Mix P, SERVA Electrophoresis). The samples were then subjected to SDS-PAGE under reducing conditions. IGFBP-3 proteolysis was analyzed by Western blot. The experiments were performed in triplicate.

### Scratch-Repair Assay

To evaluate the effect of IGFBP-3 on cell motility and migration, a scratch-wound assay was done on WM793, Me501 and B16 cells. Monolayer cells were scraped with a pipette tip to generate a scratch wound after cells reached confluence. The wounded surface was washed with 1×PBS and incubated in RPMI without fetal bovine serum in the presence or in the absence of recombinant IGFBP-3 (ABd serotec). Cell migration into the wound was monitored by phase microscopy using an Axiovert 200 M microscope with digital camera (Carl Zeiss, Thornwood, NY), taking pictures at 2, 4, 6, 24, and 48 h. The images were captured by AxioVision 4.0 software (Carl Zeiss). The closure of the initial gap area was assessed by calculating the difference between the initial and the remaining wound area at each time point. All the experiments were repeated at least three times.

### Bd Biocoat Matrigel Invasion Assay

BD matrigel chambers, stored at −20°C, were kept at room temperature for 1 h, after which 500 µL of RPMI were added to the wells. The inserts were then transferred to the medium-containing wells. RPMI (500 µL) was added to the inserts and kept at 37°C in an incubator with 5% CO_2_ for equilibration. After 2 h, the medium in the inserts was aspirated and inserts were placed into the wells containing RPMI and 5% FBS. Overall, 50000 cells in 500 µL of RPMI were added to the inserts. The plates were incubated for 22 h in a CO_2_ incubator at 37°C. The chamber inserts were then stained using the Diff-Quick staining kit (Dade-Behring) according to the manufacturer's instructions. Finally, the membranes were separated with a sterile scalpel and observed using a light microscope. The number of cells that had passed through the membranes were counted as a measure of their migration potential. All the experiments were repeated at least three times.

### Tyrosinase Activity Assay and L-Dopa Staining

The effect of IGFBP-3 on tyrosinase activity was measured both in the absence and in the presence of 2% serum. The cells were treated with IGFBP-3 for different times (24, 48 and 96 h). After treatment, the cells were washed in ice-cold phosphate-buffered saline (PBS), lysed in 0.1 M phosphate buffer (pH 6.8) containing 1% Triton X-100 and complete protease inhibitor cocktail (Roche Diagnostics, Sydney, NSW, Australia) for 20 min. at 4°C, then clarified by centrifugation (13,000/g, 5 min.). Total protein concentration in the cell lysates was estimated by the Bradford assay. The tyrosinase activity was determined by measuring the rate of L-DOPA oxidation. 100 µL aliquots of the cell extracts were incubated with 100 µL L-DOPA (3 mg/mL in 0.1 M phosphate buffer, pH 6.8) at 37°C for up to 24 h. The absorbance at 490 nm of each sample was measured and normalized over the total protein concentration in the sample. The tyrosinase activity was calculated as the ratio between the normalized 490 nm absorbances of IGFBP-3 treated and untreated samples.

### Melanin Measurement

Melanoma cells were harvested, washed with PBS, counted, lysed in 0.5% Nonidet-P40/PBS. Melanins were solubilized in 0.1 M sodium hydroxide. The optical density of the clear supernatants was measured at 475 nm. Melanin content is expressed as micrograms/milligram total protein using a standard curve generated using synthetic melanin (Sigma).

### Glycemic Levels

Glucose levels of IGFBP-3 treated and untreated mice were measured every three days from the start of the experiment with the glucose meter Accu-Chek, Aviva System (Roche), taking a drop of peripheral blood.

### Cell Cycle Analysis

Cells were treated with IGFBP-3 for 24 h. After treatment the cells were fixed in methanol-acetone 4∶1 v/v and stained with 20 mg/mL propidium iodide (Sigma) for 30 min at room temperature in the presence of 40 mg/mL RNAse A (Roche). DNA content was evaluated by cytofluorimetry with an Epics XL analyser (Coulter Corporation).

### *In Vivo* Tumor Growth Analyses

Female CB.17 SCID/SCID mice aged 4–5 weeks (Harlan; Correzzana, Milan, Italy) were kept under specific pathogen-free conditions and fed *ad libitum*. Mice were housed in micro-isolator cages, and all food, water, and bedding were autoclaved prior to use. Each mouse was injected subcutaneously in the right flank with 0.5×10^6^ human melanoma Me501 cells that had been resuspended in 0.2 mL of RPMI-1640 containing 10% FCS. Three days after tumor injection IGFBP-3 resuspended in normal saline was administered intra-peritoneally (i.p.) three times a week with one day interval, at the doses of either 0.37 or 1.87 mg/kg. Tumor dimensions were measured three times per week with calipers. Tumor weight was estimated according to Geran et al. using the following formula: tumor weight (mg)  =  length (mm) × width^2^(mm)/2 [22].

At least 8 mice were used for each treatment group. Data are expressed as the mean value of tumor weight with 95% confidence intervals. Mice were monitored for the duration of the *in vivo* experiments for glycemia, body weight, hair ruffling, and the presence of diarrhea. All mice were killed at the end of the experiments, 28 days after the injection of the human tumor cells.

### Ethic Statement

Animal experiments were conducted in strict accordance with the recommendations in the Guide for the Care and Use of Laboratory Animals of the National Institutes of Health. The protocol was approved by the Committee on the Ethics of Animal Experiments of Istituto Superiore di Sanità, Italy. All surgery was performed under sodium pentobarbital anaesthesia, and all efforts were made to minimize suffering.

### Statistical Analysis

In order to establish which variables were statistically related to high IGFBP-3 serum level in melanoma patients, data were entered into a Microsoft Excel spreadsheet and analyzed by the ANOVA test; a P value <0.05 was considered statistically significant (Table 1).

**Table 1:** Correlation between IGFBP-3 serum level and patients' recorded variables.

Patient Characteristics (N = 48)	No. of Patients(Mean value of IGFBP-3 in ng/ml)	ANOVAP value
SexMaleFemale	25 4780.751723 4881.1966	P = 0.744
Age, years≤4041–60>60	7 5193.534417 4825.578224 4724.8643	P = 0.592
Breslow's thickness, mm≤1.001.01–2.002.01–4.00>4.00Unknown	9 5372.41647 5026.741217 4353.584712 5059.64023 4506.9185	P = 0.130
UlcerationAbsentPresentUnknown	20 5026.215225 4709.65013 4506.9185	P = 0.529
Melanoma histotypeSSMNMALMUnknown	24 5173.468920 4521.59901 3670.32563 4506.9185	P = 0.118
DeathNoYes	36 4970.938012 4402.7122	P = 0.105
Disease presenceat the time of sample withdrawalNoYes	32 4982.495116 4521.6544	P = 0.153
ProgressionNoYes	26 5054.492422 4562.2505	P = 0.106
Disease stage at the time of sample withdrawalStage 0Stage 1Stage 2Stage 3Stage 4	010 5252.908015 5040.53629 5108.488514 4119.4853	P = 0.020
Metastatic volume*0–15cc≥ 15cc	36 5051.485812 4161.0686	P = 0.009

Abbreviations: IGFBP, insulin-like growth factor binding protein; SSM, superficial spreading melanoma; NM, nodular melanoma; ALM, acral lentiginous melanoma.

^*^A receiver operating characteristic (ROC) curve analysis was used to find an average value of metastatic volume in order to dichotomize the patients into two subgroups. Each measurable metastases, measured according to RECIST guideline, was considered as a spherical formation and the volumes calculated using the following formula: volume  =  4/3 π r^3^.

A receiver-operating-characteristic (ROC) curve analysis was used to find an average value of metastatic volume, IGFBP-3 serum level and IGF1/IGFBP-3 molar ratio, in order to dichotomize the patients into two subgroups, according to the cut-off obtained.

Overall survival (OS) was calculated as the time lasting from the first sample withdrawal to the date of death or last follow-up; the OS curves were compared using the Kaplan-Meier method in order to correlate the survival time with IGFBP-3 serum level and IGF1/IGFBP-3 ratio.

Statistical analysis for immuno-histochemical results was conducted with the Mann Whitney U test. To assess significant correlations, the Spearman correlation coefficient was calculated.

A *t* test analysis was performed to compare the mean tumor volumes in xenografted SCID mice.

P values <0.05 were regarded as statistically significant.

Statistical analyses were performed through MedCalc® version 12.1.4.0 [23].

## Results

### IGFBP-3 Serum Levels Correlate with Survival in Melanoma Patients

In a previous work, we showed that serum levels of IGFBP-3 are lowered significantly in stage-IV melanoma patients [15]. Here, we have confirmed and extended those results, examining a larger number of patients and following over time a number of them. IGFBP-3 and IGF-1 serum levels were estimated respectively by ELISA and RIA measurements; the data obtained were correlated with various parameters relative to the patients by the ANOVA methodology.

As shown in Table 1, seric IGFBP-3 levels correlated significantly with (i) disease stage at the time of sample withdrawal (P = 0.02) and (ii) metastatic volume (P = 0.009).

**Figure 1:**
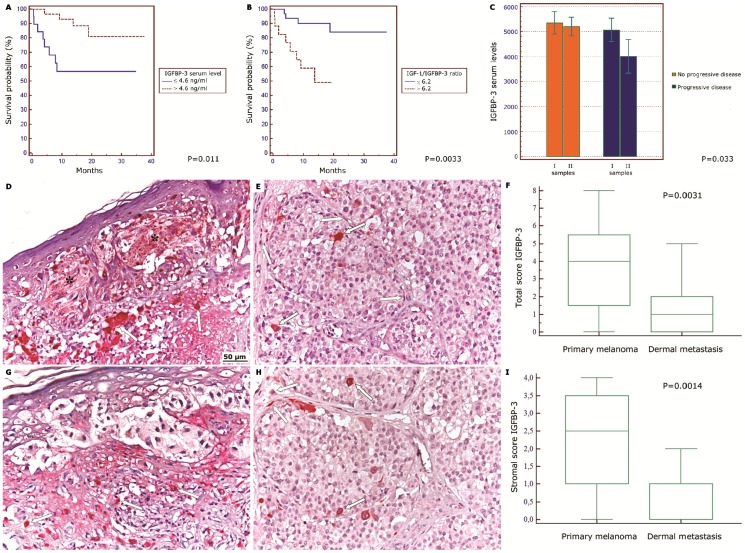
Low IGFBP-3 levels correlate with melanoma progression and patients' survival. (A) Kaplan-Meier survival curve of melanoma patients having seric IGFPB-3 concentration higher (red line) or lower (blue line) than 4.6 ng/mL. Cut-off value was chosen by ROC curve. P value was calculated by log rank test. (B) Kaplan-Meier survival curve of patients with IGF-1/IGFBP-3 ratio higher (red line) or lower (blue line) than 6.2. Cut-off value was chosen by ROC curve. P value was calculated by log rank test. (C) Mean IGFBP-3 serum levels measured twice (28 patients) during the follow-up. Orange boxes: Patients with stable disease. Blue boxes: patients with progressive disease. P value was calculated by ANOVA test. (D,G) IGFBP-3 immuno-staining in tissue samples from primary melanomas. (E, H) IGFBP-3 immuno-staining in tissue samples from metastatic melanomas. The asterisks indicate positive melanocytes nests in primary melanoma. The arrows indicate IGFBP-3 positive stromal cells. Original magnification, X100. (F,I) Graphic data processing for total (upper panel) and stromal (lower panel) IGFBP-3 immuno-staining score. 20 different samples were taken in each case and the number of IGFBP-3 positive cells was separately counted by two researchers. For each slide, at least 7–10 microscopic fields were randomly chosen. The immuno-histochemical score was evaluated as described in the Methods separately for melanocytic and stromal cells and expressed as tumour and stromal score respectively. A total score was derived for each sample as the sum of tumour and stromal score. Central boxes correspond to values from lower to upper quartile (25th–75th percentile). Middle lines show median. Vertical lines extend from minimum to maximum value. P value calculated by Mann-Whitney U test.

After normalizing for sex and age, IGFBP-3 levels were also significantly correlated with survival. 56.7% of the patients with low IGFBP-3 survived for at least 18 months, compared to 88.3% of those with high IGFBP-3 (P = 0.011) (Fig. 1A). The same was true for the ratio IGF1/IGFBP-3. 49% of patients with a higher ratio survived for 18 months, compared to 90.1% of those with a lower ratio (P = 0.0033) (Fig. 1B). In contrast, IGF-1 levels were not correlated with either disease stage or survival.

In a panel of 28 patients, the IGFBP-3 serum levels were measured twice during the follow-up. ANOVA testing revealed that the patients showing no disease progression had stable IGFBP-3 levels, while those with progressive disease displayed a significant lowering of seric IGFBP-3 (20% on average) between the first and the second sample withdrawal (Fig. 1C). Upon the whole, the present data confirmed and reinforced our previous surmise that melanoma progression is significantly correlated with loss of seric IGFBP-3. Moreover, low IGFBP-3 was highly correlated with survival and metastatic volume, suggesting that lack of this protein in the blood accompanies, and perhaps favours, metastatic dissemination of melanoma.

### IGFBP-3 in the Tissutal Microenvironment

**Figure 2:**
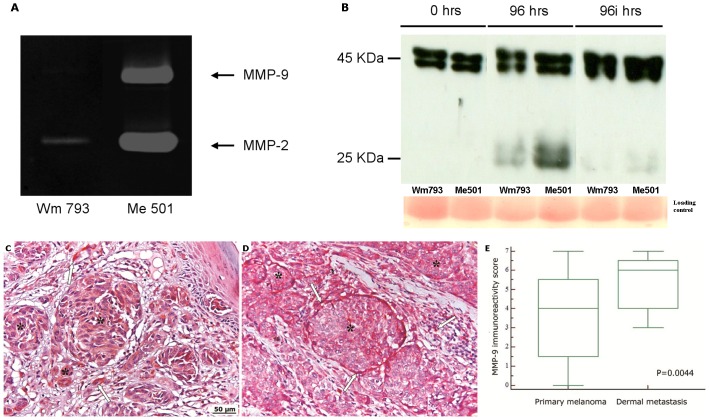
Different expression of MMP-9 in primary and metastatic melanoma. (A) Detection of MMP-2 and MMP-9 activity in the culture media of WM793 and Me501 cells by zymographic assay. The arrows point to the areas of degradation of the gelatine matrix produced by the indicated proteases (B) Western blot analysis showing degradation over time of IGFBP-3 by melanoma-produced proteases. Human recombinant IGFBP-3 was incubated for the indicated times with the culture media of the primary melanoma line WM793 or of the metastatic line Me501, in the absence (96 hrs) or in the presence (96i hrs) of protease inhibitors. A Red Ponceau staining of the original gel is shown on the bottom as the loading control. (C,D) MMP-9 immunostaining of primary (C) and metastatic (D) melanoma. Asterisks indicate melanocytes nests and the arrows indicate stromal cells. Original magnification, X100. (E) Comparison of the MMP-9 reactivity score of primary and metastatic melanoma. Central boxes represent values from lower to upper quartile (25th–75th percentile). Middle lines represent median. Vertical lines extend from minimum to maximum value. P value was calculated by Mann-Whitney U test. The reactive cells were counted, and the scores determined, as described in Fig. 1 and in the Methods section.

The progression of primary melanoma to metastatic disease may be influenced by the IGFBP-3 in the tissutal microenvironment. Most of the circulating IGFBP-3 is produced by the liver; however, the protein is also secreted by several other inflammatory and mesenchymal cell types and by melanocytes themselves. Notably, an inverse correlation has been observed between the amount of IGFBP-3 produced by melanoma tissue and the metastatic capacity of the cells [24]. To evaluate the presence of IGFBP-3 in the immediate environs of the tumour, immuno-histochemical analyses of primary and metastatic tumour samples taken from patients were performed. As shown in Fig. 1 (D,G), a diffuse intracellular IGFBP-3 staining was observed in primary melanomas as well as in peri-tumoral stromal cells including monocytes/macrophages, lymphocytes, granulocytes and fibroblasts. In metastatic tumours IGFBP-3 staining was much weaker or absent (Fig. 1 E,F,H,I). Previously, we hypothesized that IGFBP-3 loss in both blood and tissues could be accounted for by degradation by tumour-produced proteases. This was further investigated. Firstly, secretion of proteases by cultured primary and metastatic melanoma cells was evaluated by zymography. As shown in Fig. 2A, the primary melanoma line WM793 secreted only small amounts of a gelatinase (presumably metalloprotease-2) (MMP-2). By contrast, the metastatic cells Me501 produced large amounts of proteases, especially MMP-2 and metalloprotease-9 (MMP-9). Accordingly, the culture media of metastatic, but not primary, cells caused extensive degradation of recombinant IGFBP-3 in vitro. (Fig. 2B). This result agrees with our previous finding that recombinant IGFBP-3 was degraded upon incubation with blood serum from stage-IV melanoma patients [15].

**Figure 3:**
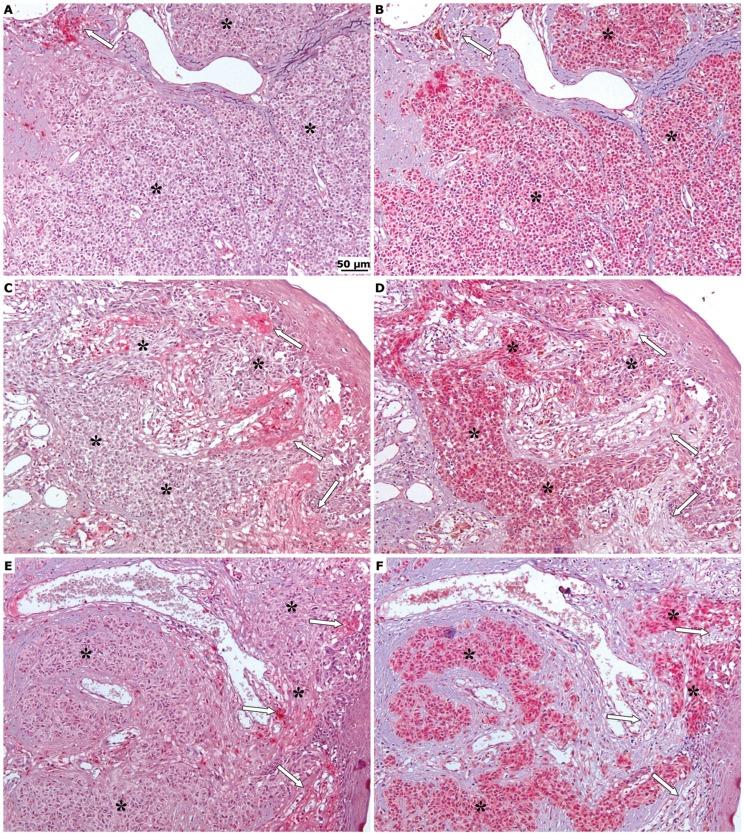
Inverse correlation between immuno-histochemical expression of IGFBP-3 and MMP-9. Comparison of the immunohistochemistry for IGFBP-3 (A, C, E) and MMP-9 (B, D, F) in sequential sections obtained from primary melanomas and dermal metastases. The asterisks mark the melanocytes nests while the arrows indicate the stromal tissue. Original magnification X100. The IGFBP-3 and MMP-9 reactive cells were counted, and the scores determined, as described in Fig. 1 and in the Methods section.

Secondly, tumour samples taken from patients were immuno-stained for both IGFBP-3 and MMP-9. As shown in Fig. 2 (C,D,E), the MMP-9 immunoreactivity of both tumor and stromal cells was significantly higher in metastatic lesions than in primary tumors. Strikingly, there was an almost perfect inverse correlation between the areas of low or no IGFBP-3 staining and those of high MMP-9 staining (Fig. 3), suggesting that the enzyme could indeed have a role in the degradation of IGFBP-3.

### IGFBP-3 Inhibits Motility and Invasion in Cell Cultures of Metastatic Melanomas

To investigate the cellular action of IGFBP-3, cultured cell lines of primary and metastatic human melanoma (WM793 and Me501) and of metastatic murine melanoma (B16F10) were subjected to scratch-repair and trans-well migration/invasion tests in the absence and in the presence of IGFBP-3.

**Figure 4:**
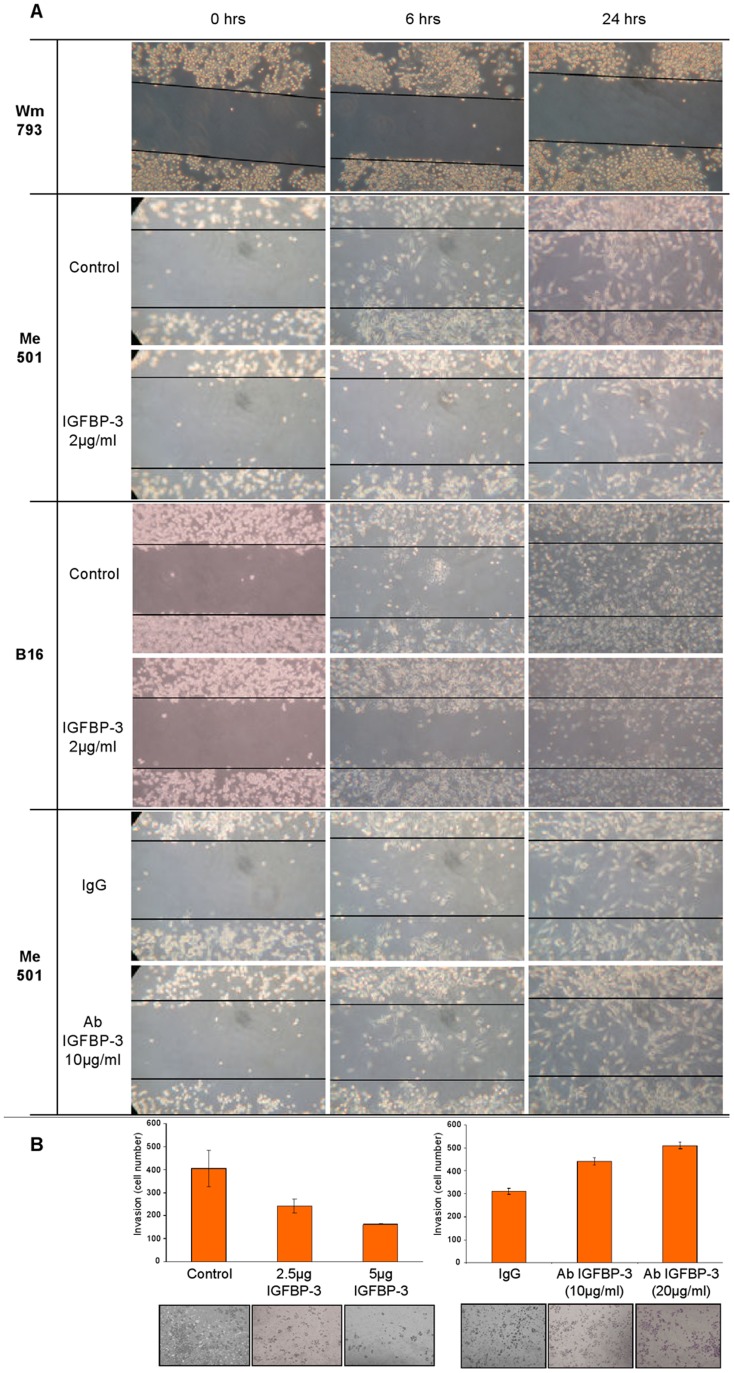
IGFBP-3 reduces motile and invasive behavior of melanoma cells. (A) First row: a representative migration pattern of human primary melanoma cells (WM793), either IGFBP-3-treated or untreated. Second and third row: migration pattern of human metastatic melanoma cells (Me501) untreated or treated with 2 µg/mL IGFBP-3. Fourth and fifth row: murine metastatic melanoma cells (B16), untreated or treated with 2 µg/mL IGFBP-3. Last two rows: Me501 cells treated with unspecific IgG or with 10 µg/mL of anti-IGFBP-3 antibody. Scratch-test assays were performed as described in the Methods and the images were captured at 0, 6, 24 h after incubation. All the experiments shown were repeated three or more times. (B) Left panel: invasiveness pattern of Me501 cells, untreated or treated for 48 h with the indicated doses of IGFBP-3. Right panel: invasiveness pattern of Me501 cells untreated or treated for 48 h with the indicated doses of anti-IGFBP-3 antibodies. Trans-well migration/invasion assays were performed as described in the Methods. Invasiveness assays were repeated three or more times.

For scratch-repair analysis, primary (WM793), and metastatic (Me501 and B16F10) melanoma cell lines were grown to confluence in 10% serum. After introduction of the scratch, the cells were supplemented with serum-free medium without or with added IGFBP-3 (2 ug/mL) and followed for up to 48 hours. As shown in Fig. 4A (first row), primary melanoma cells were scarcely mobile and did not appreciably migrate into the scratch; this motility pattern remained unchanged upon treatment with IGFBP-3 (not shown). By contrast, metastatic cells were highly mobile, migrating within the gap already after 6 hours and largely covering the wound in about 24 hours (Fig. 4A, second and fourth row). Strikingly, treatment with IGFPB-3 markedly retarded the migration of both human and murine melanoma cells (Fig. 4A, third and fifth row). Also, addition of anti-IGFBP-3 antibodies to the culture media, to sequester any cell-made IGFBP-3, accelerated scratch repair (Fig. 4A, last two rows).

Trans-well-migration/invasion assays revealed that the invasive capacity of metastatic melanoma cells was also strongly impaired by treatment with IGFBP-3, while being enhanced by treatment with anti-IGFBP-3 antibodies (Fig. 4B).

**Figure 5:**
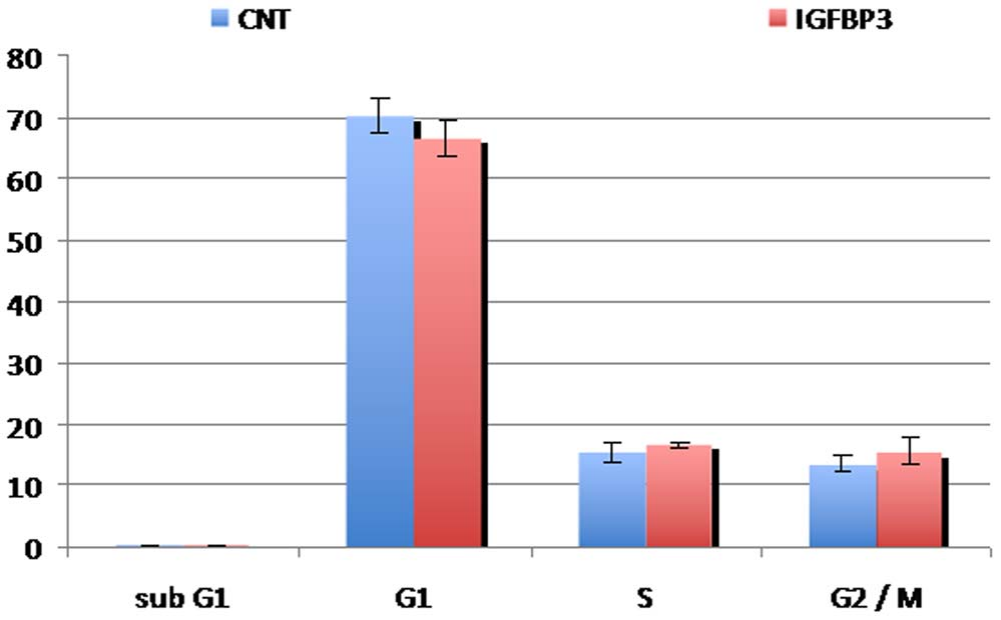
IGFBP-3 does not alter cell proliferation. Cell-cycle distribution evaluated by FACS analysis of Me501cells untreated or treated with IGFBP-3. The areas of the peaks corresponding to each phase were evaluated and plotted as an histogram. The experiment was performed in triplicate.

Importantly, the effect of IGFBP-3 on both migration and invasion was independent of cell proliferation. FACS analyses performed on semi-confluent cells did not reveal any difference in cell-cycle distribution between the IGFBP-3 treated and untreated cells (Fig. 5). As expected in dearth of serum, both groups underwent cell-cycle arrest after a while, but the timing of the arrest was unaffected by IGFBP-3 treatment. Furthermore, the amount of cells in sub-G1 was similar in treated and untreated samples, indicating that IGFBP-3 did not induce any significant apoptosis. This was confirmed by measurements of the apoptotic marker annexin-V, which remained unchanged upon IGFBP-3 treatment (not shown).

### IGFBP-3 Inhibition of Cell Motility/Invasion Is Independent of IGF-1

As the main functions of IGFBP-3 in blood serum is to modulate the bioavailability of IGFs, it was important to assess whether the anti-invasive effect of IGFBP-3 depended on IGF-1 sequestering.

We concluded that it did not, for the following reasons.

**Figure 6:**
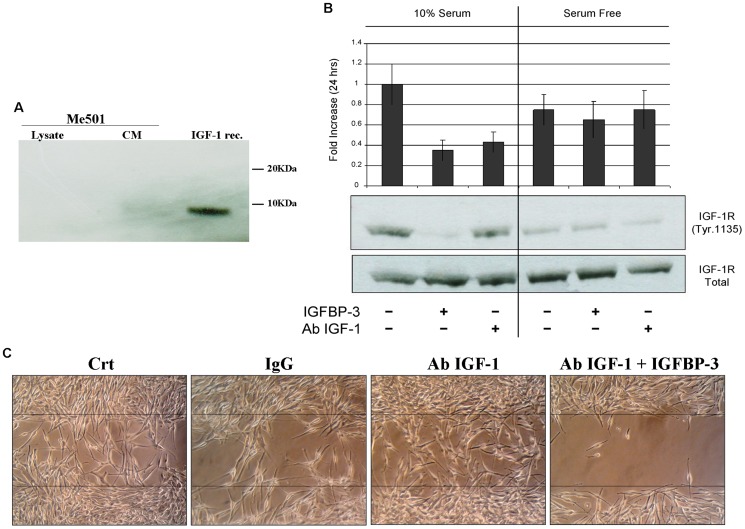
IGFBP-3 acts independently of IGF-1. A) Western blot analysis for the detection of IGF-1 in lysates or culture media (CM) of Me501 cells. IGF-1 rec represents the positive control with recombinant IGF-1 (5 ng). B) Western blot analysis for detection of Tyr 1135-phosphorylation on the IGF-1 receptor β. Me501 cells were grown for 24 h in the presence of 10% serum (left panels) or in the absence of serum (right panels). For each group, treatments with IGFBP-3 or with anti-IGF-1 antibodies were performed. The amount of phosphorylated IGF-1-β receptor was quantified by densitometric analysis using total receptor (which remained unchanged) as the internal standard. C) Scratch-repair capacity of Me501 cells in the presence of anti-IGF1-antibodies and of IGFBP-3. The images shown are representative of experiments that were repeated at least three times.

Firstly, migration and invasion assays were performed in the absence of serum and hence in the absence of IGFs. The possibility that the cells themselves produced IGF-1 was discounted on the basis of western blot analysis of both cell lysates and culture media (Fig. 6A). IGF-1 mediated effects were further excluded by determining the state of activation of the IGF-1 receptor (IGF-1R).

In the culture conditions used to perform the motility/invasiveness assays, the IGF-1R of Me501 cells was hypo-phosphorylated, hence scarcely active, and remained unaffected by treatment with both IGFBP-3 and anti-IGF-1 antibodies. Control experiments showed that the IGF1 receptor was active in the presence of serum and that under these conditions IGF-1R activity could be reduced, as expected, by treatment with both IGFBP-3 and anti-IGF-1 antibodies (Fig. 6B). Lastly, we determined whether the addiction of anti-IGF-1 antibodies to the culture medium could inhibit cell migration or contrast the anti-migratory effect of IGFBP-3. As shown in Fig. 6C, this was not the case. The ability of the Me501 cells to migrate into the wound was unaffected by anti-IGF-1 antibodies, and the latter did not influence IGFBP-3 ability to inhibit wound repair.

### IGFBP-3 Signals through the AKT Pathway

The cellular pathways involved in the response to IGFBP-3 were analysed using a phosphor-proteome profiler array. Primary and metastatic melanoma cell lines were grown to semi-confluence, transferred to a serum-free medium and treated with IGFBP-3 for 24 hours.

**Figure 7:**
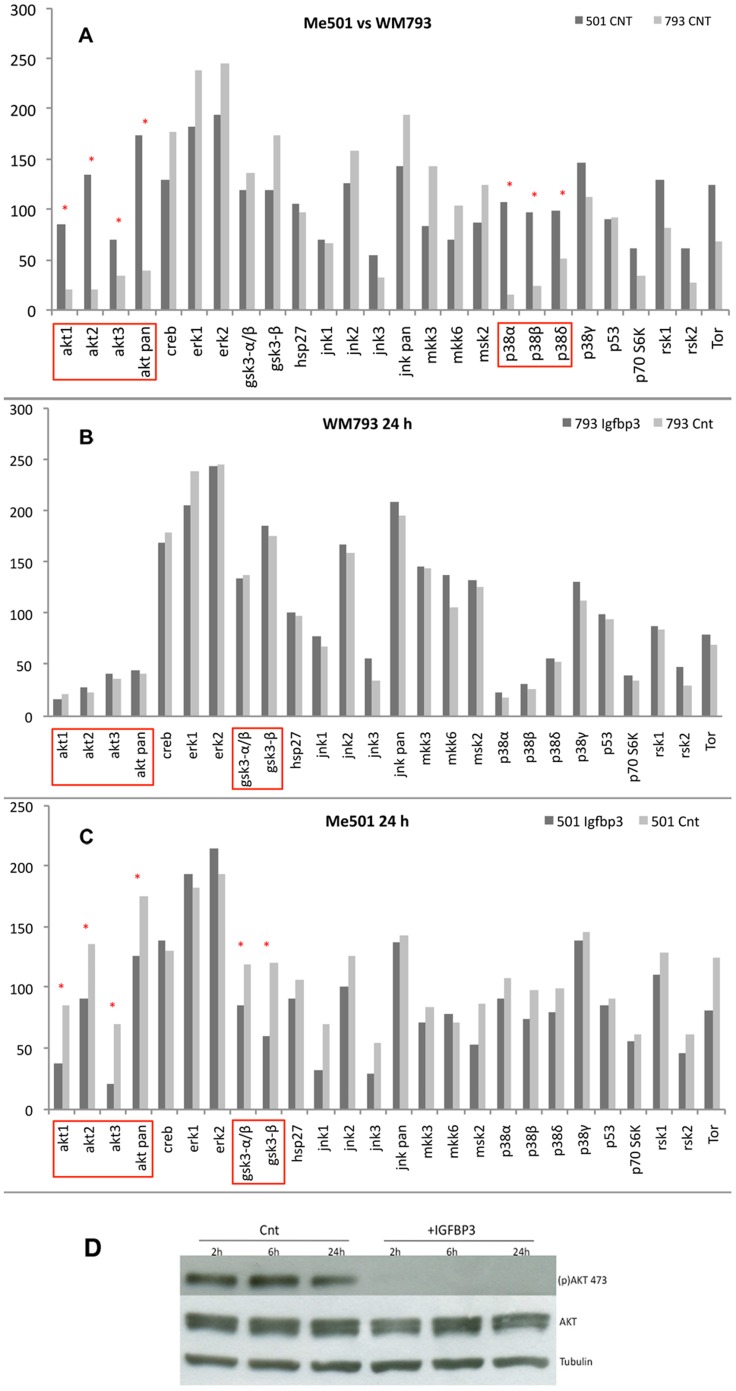
Analysis by phosphor-proteomic arrays of signal-transduction pathways involving in mediating IGFBP-3 action. (A) Phosphorylation state of the indicated markers in primary (WM 793, light grey) and metastatic (Me501, dark grey) human melanoma cells, untreated. (B) WM 793 cells before (light grey) and after (dark grey) treatment with IGFBP-3 (C) Me501 cells before (light grey) and after (dark grey) treatment with IGFBP-3. The assays were performed and quantified as described in the Methods. All determinations were repeated at least three times (D) Western blot analysis of the phosphorylation state of Akt (Ser 473) in Me501 cells untreated or treated with IGFBP-3 for the indicated times. pAKT 473 =  phosphor-(Ser473); AKT =  total AKT: Tub =  tubulin. The gel shown is representative of experiments that were repeated at least three times.

Fig. 7A shows the phosphor-proteome profiles of the primary and metastatic lines WM793 and Me501 in the absence of any treatment. As predicted by their genetic background, both lines had an active RAS-RAF-ERK pathway, confirming the central role thereof in melanoma development. However, the metastatic cells differed from the primary ones in having highly phosphorylated Akt (Ser 473) and p38 α/β kinases. Accordingly, certain downstream targets of Akt, such as GSK3β and mTOR, were also up-phosphorylated compared to the primary cell line.

Treatment with IGFBP-3 produced no effect on the WM793 cells (Fig. 7B). However, in the Me501 cells it caused a drastic inactivation of all three Akt isoforms, especially Akt3, with a concomitant dephosphorylation of GSK3β and mTOR. In contrast, the p38 kinases remained unaffected (Fig. 7C).

The activation state of Akt was further analysed at different times of treatment with IGFBP-3 (Fig. 7D). Dephosphorylation was essentially complete after 2 hours and remained so for the ensuing 24 hours. The overall amount of Akt remained unchanged throughout the experiment, indicating that IGFBP-3 had no effect on either the expression or the stability of the protein. Upon the whole, the above results suggest that (i) Akt hyper-activation is an important event in the transition between primary and metastatic melanoma, modulating the motility and invasion capabilities of the cell (ii) the inhibitory action of IGFBP-3 on cell motility requires down-regulation of the Akt pathway, probably involving GSK3β and mTOR as downstream effectors. The p38 kinases, which have been reported to stimulate the migratory and growth capacity of melanoma cells, remained unaffected by IGFBP-3 treatment.

### IGFBP-3 Induces Melanocytic Differentiation of Metastatic Melanoma Cells

**Figure 8:**
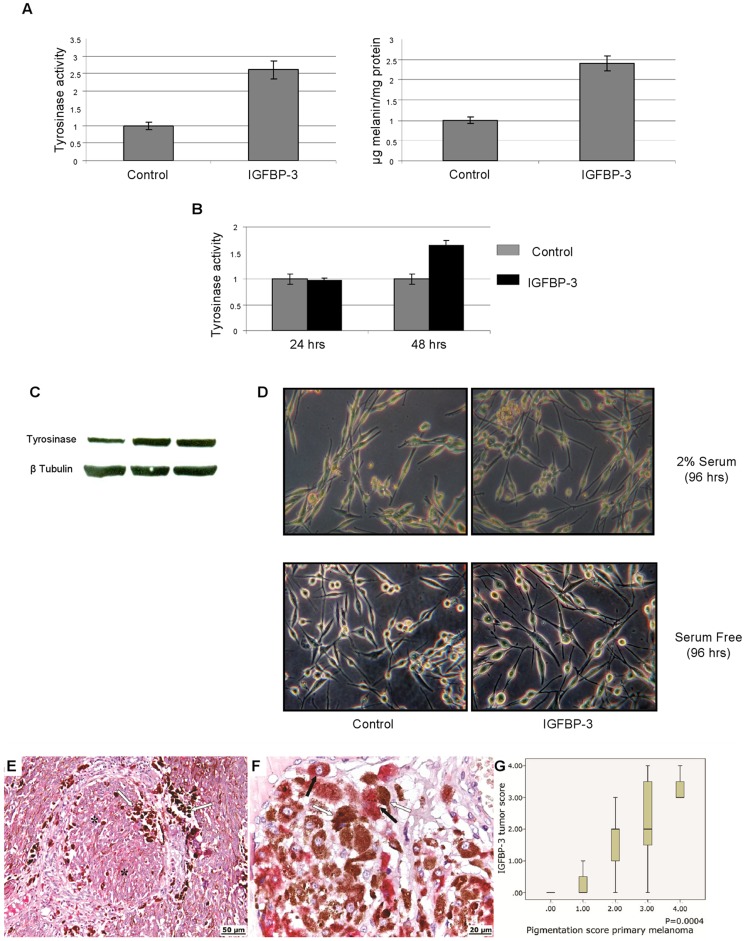
IGFBP-3 up-regulates tyrosinase activity and increases melanin content in Me501 cells. (A) Tyrosinase activity measured by L-DOPA oxidation (left panel) and melanin content determined by measuring the absorbance at 405 nm (right panel) of lysates of Me501 cells grown in 2% serum, untreated or treated for 96 h with IGFBP-3 (2 µg/mL). (B) Tyrosinase activity of lysates of Me501 cell grown in the absence of serum for 24 and 48 h, treated and untreated with IGFBP-3 (C) Western blot analysis of tyrosinase content in Me501 cells after 0, 24 and 48 h of treatment with IGFBP-3 (2 µg/mL). (D) Phase-contrast microscopy of untreated and IGFBP-3-treated (2 µg/mL) Me501 cells, grown in the presence and in the absence of serum. Original magnification X100. (E, F) IGFBP-3 immuno-histochemical positivity in primary melanoma correlates positively with melanin content. IGFBP-3 is stained red, while melanin stains as dark-brown spots (E) X200 magnification (F) X400 magnification. The asterisks mark the IGFBP-3 immuno-positive melanocyte nests, the white and black arrows indicate the clusters of melanin and IGFBP–3 positive melanocytes, respectively. (G) Box plots showing the correlation between IGFBP-3 tumor score (calculated as described in the Methods) and sample pigmentation. P value was calculated by Mann-Whitney U test.

Besides losing the ability of migrate and invade, the IGFBP-3 treated cells kept in culture for a few days acquired a more dendritic appearance and looked visibly blacker, suggesting that they were steered towards melanocytic differentiation (Fig. 8D). This behaviour is in agreement with the fact that one the downstream targets of Akt most affected by IGFBP-3 treatment was GSK3β, a factor whose activation by dephosphorylation triggers melanin synthesis and differentiation in melanocytes [25]. Accordingly, we tested whether IGFBP-3 treatment resulted in activation of the melanin synthesis pathway, by measuring the tyrosinase activity and the amount of melanin in treated and untreated Me501 cells. As shown in Fig. 8 (A,B,C), IGFBP-3 treated Me501 cells had a significantly higher tyrosinase activity and melanin content compared to untreated cells. These *in vitro* results were corroborated by immuno-staining of tumor samples taken from patients. As shown in Fig. 8 (E,F,G), only tumor tissues positive for IGFBP-3 contained significant amounts of melanin.

### *In Vivo* Anti-Tumoral Effects of IGFBP3 Treatment

**Figure 9:**
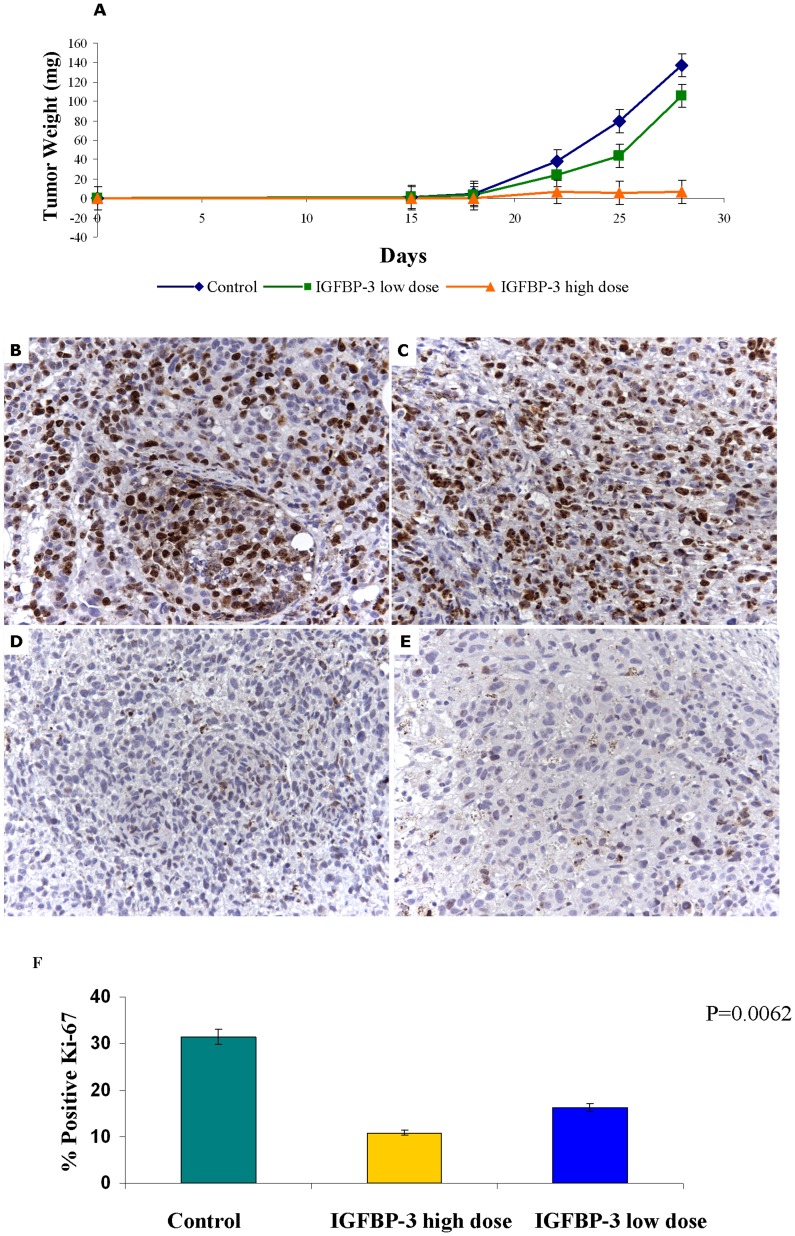
IGFBP-3 inhibits tumour growth *in vivo*. (A) Effect of treatment with low (0.37 mg/Kg, green curve) and high (1.87 mg/kg, orange curve) IGFBP-3 doses on the growth over time of Me501 xenografts in SCID mice. Mean results are representative of two different experiments (each experiment on at least 8 mice). Tumor size was measured three times per week with calipers, and volume was calculated as described in the “Methods” section. (B) Immuno-histochemical staining of the cell proliferation marker Ki-67 in Me501 xenografts in tumours excised from SCID mice untreated (A–B) or treated with 1.87 mg/kg IGFBP-3 (C–D). (F) Graph reporting the mean values of Ki-67 positive cells in melanomas from control mice and mice treated with low and high doses of IGFBP-3. The proliferation index was determined as the percentage of proliferating cells (Ki-67 positive cells) in a population of about 800 cells. For this purpose two representative fields were randomly selected in each section and blind-counted by two independent observers. P value was calculated by Mann-Whitney U test.

To assess the potential clinical relevance of the *in vitro* results, we evaluated *in vivo* the anti-tumoral activity of IGFBP-3 using SCID mice xenografted s.c. with Me501 cells. Three days after tumor injection, mice received intraperitoneal (i.p.) injections of saline, or 0.37 mg/Kg or 1.87 mg/kg IGFBP-3 three times a week (TTW) for three weeks and tumor growth was estimated during the follow-up. As illustrated in Fig. 9, the growth of Me501 tumors in mice treated with IGFBP-3 was significantly reduced as compared with control animals, with a dose-dependent efficacy, and the size of IGFBP-3-treated tumors was significantly smaller than control tumors at follow-up (*P* = 0.0005), indicating that IGFBP-3 treatment may exert an antineoplastic activity *in vivo*. More in detail, Tumor Volume Inhibition percent (TVI%) in IGFBP-3-treated mice was respectively of 40,33 and 94,61% at 28 days post-tumor implant. At sacrifice, tumor diameter was significantly lower (t-Test p<0.001) in IGFBP-3 (3±1,5 and 4.5±2 mm) in comparison to saline (9.0±3 mm) treated mice. Moreover, complete response (CR) was obtained in 2/8 mice treated with the lower IGFBP-3 concentration, in 3/8 mice treated with the higher IGFBP-3 concentration but in none of the saline group. Four weeks after tumor implant mice were sacrificed and tumors were collected for histological examination. We observed that the tumor mass of treated mice was occupied by large necrotic areas that accounted for most of the tumor size, indicating that the cytotoxic effects of IGFBP-3 were more profound than those anticipated by the simple evaluation of *in vivo* tumor size (not shown). We also noticed a marked reduction in Ki67 positive cells in the IGFBP-3-treated groups (Fig. 9). Moreover, tumors from IGFBP-3 treated mice were consistently darker than those from untreated mice, suggesting up regulation of melanin synthesis. Indeed, tyrosinase activity measured on lysates of tumour tissues was higher in the tumours excised from IGFBP-3-treated mice, confirming the observations made on cultured melanoma cells (not shown).

Melanoma-bearing SCID mice treated with IGFBP-3 did not show any signs of systemic toxicity such as weight loss, diarrhea, or hair ruffling during the dosing period until sacrifice. Considering the role of IGFBP-3 in regulating glycaemia, glycemic values were also assessed every three days after the start of treatment with IGFBP-3. No alteration in glycemic level was observed in IGFBP-3 treated mice (not shown).

## Discussion

In this study we show that IGFBP-3 has a remarkable anti-tumoral activity on malignant melanoma, both *in vitro* and *in vivo*. In agreement with a previous study [15], clinical observations revealed that IGFBP-3 serum levels of melanoma patients were highly correlated with disease stage and progression. Indeed, stage IV patients underwent a significant loss of circulating IGFBP-3, whose extent was a strong predictor of survival time. The same relationship held for the ratio IGF-1/IGFBP-3, while the IGF-1 levels *per se* did not correlate with melanoma stage, suggesting that the apparently protective function of IGFBP-3 is not limited to preventing IGF-1 from stimulating cell proliferation.

Remarkably, seric IGFBP-3 levels were also strongly correlated with metastatic volume and with patients' survival. This may suggest that melanomas have an active role in destroying IGFBP-3, and indeed many tumors are known to produce proteases. In fact, we show here that the IGFBP-3 content of melanomas and their tissutal environment becomes progressively lower with advancing disease, and that IGFBP-3 loss is accompanied by a parallel increase in MMP-9. Accordingly, metastatic, but not primary, melanoma cells secreted large amounts of a MMP, presumably MMP-9. These results agree with a former study reporting that blood serum from IV-stage melanoma patients was uniquely able to degrade IGFBP-3 in vitro [15]. It is possible, therefore, that melanomas ingrain a self-sustaining loop, destroying IGFBP-3 as they grow and becoming as a consequence ever more aggressive. However, the alternative hypothesis that melanomas inhibit tissutal, or even hepatic, production of IGFBP-3 cannot yet be discounted and is currently being tested.

*In vitro* assays on cell cultures revealed that IGFBP-3 affects the cells' ability to migrate and invade rather than that to proliferate. The WM793 primary-melanoma cells, which have a relatively high proliferation rate but are scarcely mobile or invasive, did not respond to IGFBP-3 either at the phenotypic or at the molecular level. In contrast, the highly motile and invasive behavior of the two cell lines of metastatic tumors analyzed (Me501 and the murine line B16) was strongly inhibited by treatment with human recombinant IGFBP-3. Notably, the effect of IGFBP-3 on Me501 and B16 cells was the same despite the fact that the former line has a mutated form of NRAS, while the latter has this gene in a wild-type state (see Methods).

Phosphor-proteome profiling of certain signal transduction pathways involved in tumoral transformation revealed that the metastatic cells, unlike the primary ones, had active Akt and p38 kinases. Treatment with IGFBP-3 turned off Akt, while p38 kinases remained unaffected. Akt inactivation caused the concomitant dephosphorylation of certain targets thereof, notably GSK-3β and mTOR. That GSK-3β in particular may be a mediator of the anti-metastatic effect of IGFBP-3 is suggested by the fact that IGFBP-3-treated cells showed a tendency to revert to a more differentiated melanocytic phenotype, as indicated by the increase of both tyrosinase activity and melanin content. In fact, GSK-3β is known to promote melanin synthesis and melanocyte differentiation [25]. Work is in progress to unravel the molecular mechanism of action of IGFBP-3.

The inhibitory effect of IGFBP-3 on motility and invasiveness appears to be independent of IGF-1. This is borne out by the facts that the motility/invasiveness assays were performed in the absence of IGF-1, that the cells under study did not produce IGF-1 and had the IGF-1 receptor in an inactive state. Indeed, it is well known that IGFBP-3 has a biological activity of its own on a variety of tumor cell lines. IGFBP-3-induced proliferation arrest and apoptosis induction were described by other investigators on tumors other than melanoma [26]–[27], but to the best of our knowledge inhibition of cell motility and invasiveness has not been reported previously. Future work will clarify whether this is specific of melanoma or can be extended to other tumors.

The anti-tumoral effect of IGFBP-3 was also investigated *in vivo* using a murine model, namely SCID mice which were inoculated with human melanoma (Me501) cells. Tumor growth was strongly inhibited already upon administering low doses of IGFBP-3, and completely arrested in 2 out of 8 cases already at lower doses, and in 3 out of 8 cases at higher doses. The tumors excised from treated mice had significantly lower levels of the mitotic marker Ki-67, indicating that IGFBP-3 exerted a certain anti-proliferative activity in vivo, most probably due to the sequestering of IGF-1. However, the IGFBP-3-induced reversion of tumor cells towards a melanocytic phenotype observed *in vitro* was also apparent *in vivo*, since tumors from treated mice appeared darker and had higher tyrosinase activity. Work is in progress to specifically assess the *in vivo* effect of IGFBP-3 on melanoma metastatic spread.

Importantly, IGFBP-3 did not produce evident toxic effect even at the higher doses, nor had detectable negative impact on glucose metabolism, indicating that it might be safely used as a therapeutic agent.

Altogether, the results presented here suggest that IGFBP-3 is a potentially interesting anti-tumoral agent, all the more because it is a physiological factor that is not expected to have major adverse effects when given therapeutically. At the least, it may qualify for a valid adjuvant therapy in melanoma (and perhaps other cancers) during treatment with conventional anti-tumoral drugs.
